# Multidetector computed tomography for patients with congenital heart disease: a multi-center registry from Africa and Middle East; patients’ characteristics and procedural safety

**DOI:** 10.1186/s43044-021-00217-x

**Published:** 2021-10-16

**Authors:** Dina Adel Ezzeldin, Mohamed Saber Hafez, Amr Mansour

**Affiliations:** Cardiology Department, Faculty of Medicine, Ain Shams University, 38 Abbaseya Square, Next to Alnour Mosque, Cairo, 11591 Egypt

**Keywords:** Congenital heart disease, Cyanotic heart disease, Multidetector computed tomography, Radiation safety, Registry

## Abstract

**Background:**

We aimed to establish a clinical registry for patients with congenital heart disease who referred to multidetector computed tomography in our country, to describe the pattern and clinical profile of such patients and document the safety and efficacy of the procedure in our daily practice.

**Results:**

A total 2310 studies were analyzed after excluding studies with missed, and lost data. Half of our study population—1215 patients—52.5% were males. The median age of the patients was 12 months (IQR 37 months), and the youngest patient was 3 days old. The eldest patient was 50 years old. 68.27% of the patients were less than 2 years old, and two-third of the whole studied population 66.7% had cyanotic heart disease. Minor local access complications, complications related to anesthetic drugs, and allergic reactions were the most commonly encountered complications, with only single mortality mainly due to multiple associated multisystem congenital malformation.

**Conclusions:**

Most of our patients with congenital heart disease referred for MDCT study were infants and young children. The majority of them had complex cyanotic heart disease. The study is safe, with excellent diagnostic yield and safe with very low incidence of complications.

## Background

Although echocardiography is considered the initial diagnostic modality for patients with suspected congenital heart disease, in some patients this modality can be limited in its ability to delineate extracardiac structures, and some intracardiac anomalies. Multidetector computed tomography (MDCT) has an excellent anatomic and functional assessment capabilities [1, 2], most of the studies are performed to answer specific anatomic questions raised by an inconclusive echocardiographic or angiographic evaluation [1, 2].

Despite the expanding role of cardiac magnetic resonance imaging (CMR) in congenital heart diseases, with proven outstanding superiority to yield functional and anatomical data, its use is rather limited in our country due to unavailability in many hospitals and centers, longer duration of the study, special need of MRI compatible anesthetic and monitoring equipment, cost and lack of expert personnel to perform and interpret the examinations.

Radiation exposure is the main concern in the MDCT; however, with the rapid advancement of technology, the study can be performed with very low doses of radiation and excellent images quality [3, 4].

## Methods

### Aim

To establish a clinical registry for patients with congenital heart disease (CHD) referred to perform a multidetector computed tomography, aiming at description of the pattern and clinical profile of such patients and describe the safety of this imaging modality.

### Patient’ categorization

As the MDCT study was done to address specific question and evaluate certain structure, we thought to categorize the patients in this registry according to the aim of the structure to be evaluated [3, 4].

### Image acquisition and study protocol

This is a retrospective registry, and we collected and analyzed a total number of 2458 MDCT examination of patients with congenital heart diseases who were referred from all of the country governorates to perform MDCT in the participating MDCT cardiac centers; International cardio scan, Dar Elshefa hospital and Watany scan imaging centers—between 2016 and 2019.

MDCT study was performed using a 128 detector GE Optima 660 machine. Acquisition of the images was done according to a low-dose radiation protocol with dose modulation and dose reduction to minimize radiation exposure as low as possible according to the guidelines of image acquisition recommended by the society of cardiovascular computed tomography while maintaining the images quality [4].

The image acquisition was retrospective, with electrocardiogram (ECG) gating in all patients.

The software used allowed us to delete any extrasystole beats, insert a non-detected R-peak marker, and to shift a R-peak marker to adjust for any arrhythmia occurred during acquisition of images. Other advantage of the retrospectively the ECG-gated helical acquisition technique is the ability to reconstruct data from multiple cardiac phases throughout the cardiac cycle.

The standardized scan length in our protocol was variable according to the patient’s diagnosis and the aim of the study, but in the standard design, it involved scanning of the patient’s body starting from the root of the neck till the second lumbar vertebrae in order to include the arch vessels, the liver, the upper portion of the kidneys, and the spleen to determine the patient’s situs.

All the indications of the studies were revised before acquisition of the images, and the scan length was modified when needed to be set at the minimum length clinically necessary to further reduce the radiation’s dose to maintain the ALARA principle (as low as reasonably achievable).

We ensured that the algorithms of dose optimization were followed, reviewed and revised at least annually in all of these imaging centers.

A 25 mAs were used for patients younger than three years; 80 mAs were used in patients’ weight ranging from 25 to 55 kg, and 100–140 mAs were used for patients weighing more than 55 kg.

We used 60 kV in patients weighing less than 45 kg; and 100–120 kV for patients with body weight more than 45 kg. The slice interval was adjusted at 0.625 mm interval and 0.625 mm slice thickness.

Other advantages of ECG gating acquisition are the ability to reconstruct of the images to overcome motion and breathing artifacts, and hence avoiding unnecessary repetition of the study.

We did not administer any medication to control heart rate before the study in most of the patients with the exception of very few selected cases where detailed analysis of the coronary arteries was required, such as cases of coronary artery anomalies affecting its origin and course, cases with vasculitis and suspected aneurysms with or without inside thrombi or patients with coronary fistulae.

Radiation dose in our registry was recorded in Dose length product (DLP), which is measured in mGy*cm. It is a measure of CT tube radiation output/exposure. The mean value of DLP in our registry was 513.76 mGy*cm ± 417.49).

### Contrast material

Nonionic contrast medium was given intravenously with power dual injector. Contrast and saline injections were done using medrad stellant dual head injector.

The rate of contrast injection was determined according to the size of the venous access in gauges and patients’ weight in kg, and it was calculated in mL/s ranging from 1.2 to 5.5 mL/s and followed by a saline chaser.

According to the aim of the study and patient’s diagnosis, the standardized injection of the contrast which was followed by saline chaser was sometimes modified into a mixture of 50% contrast material and 50% of saline, or 70% contrast, and 30% saline simultaneous injection to ensure adequate vessels opacification without layering and contrast-induced artifacts.

The dose of the non-ionic iso- or low osmolar contrast media used was 1–2 mL/kg in pediatric age group. The iodine concentration was 240–320 mg/mL, and the bolus tracking marker was placed on the anatomic site of clinical relevance.

### Patient’s preparation

Patients were required to fast for 4–6 h for solid food and 3 h for clear fluids before the procedure.

Kidney function test was checked in all patients before the study, with calculation of the estimated glomerular filtration rate.

### Study acquisition team

All the studies were attended by an experienced cardiac anesthesiologist, and a congenital heart disease cardiologist.

Obtaining an intravenous access for patients who were referred to perform the study on an outpatient basis was done using an appropriately sized cannula ranging from 24 Gauge to 18 gauge according to the patient’s body weight and the aim of the study. Only in exceptional cases, two simultaneous accesses were obtained in the upper limb and the lower limb.

### Safety precautions during the study

Continuous monitoring of the contrast material and saline injection pressure in Psi (pressure square inch) during the injection was done in all patients to detect any sudden rise in the pressure that may indicate kinking of the connection line between the pump and the patient, or rupture of the vein and extravasation of the contrast material. Immediate interruption of injection was done in these situations.

The room was fully equipped with all of the emergency equipment, crash car, and oxygen source.

All medications to deal with and mange any cardiac emergencies-up to cardiac arrest-, and severe allergic reaction were available.

### Anesthetic medications

For neonates, infants, and young children, all the studies were performed under sedation using a body weight adjusted dose of intravenous dexmedetomidine (1 μg/kg) over 15 min, followed by 1 mg/kg of ketamine infused slowly over 15 min, some patients received body weight adjusted intravenous Ketamine, or intravenous propofol.

The selection of the administrated drug was left mainly to the discretion of the cardiac anesthesiologist. All the patients were on spontaneous breathing with oxygen mask, unless they were transferred from the intensive care unit already on portable mechanical ventilation. All patients had continuous Electrocardiogram (ECG) and oxygen saturation pulse oximetry monitoring.

Elderly patients performed the MDCT study without anesthesia.

### Patient monitoring and recovery

All patients were transferred to a recovery room after the study for monitoring, a specialized trained nurse attended all the exams, and she was responsible for the patient’s monitoring and follow-up after the study.

### Images analysis

Analysis of the multi-planar reconstructions (MPR) images in the three orthogonal view-axial, coronal and sagittal- and in oblique views, maximal intensity projection images (MPI), and three-dimensional volume rendering reconstruction images (VRI) were performed in all of the studies.

### Statistical analysis

Data were recovered, tabulated, and entered to the Statistical Package for Social Science (IBM SPSS) version 23. *P* > 0.05 was considered non-significant, and *P* < 0.05 was considered significant.

## Results

### Demographic data of the patients

**Fig. 1:**
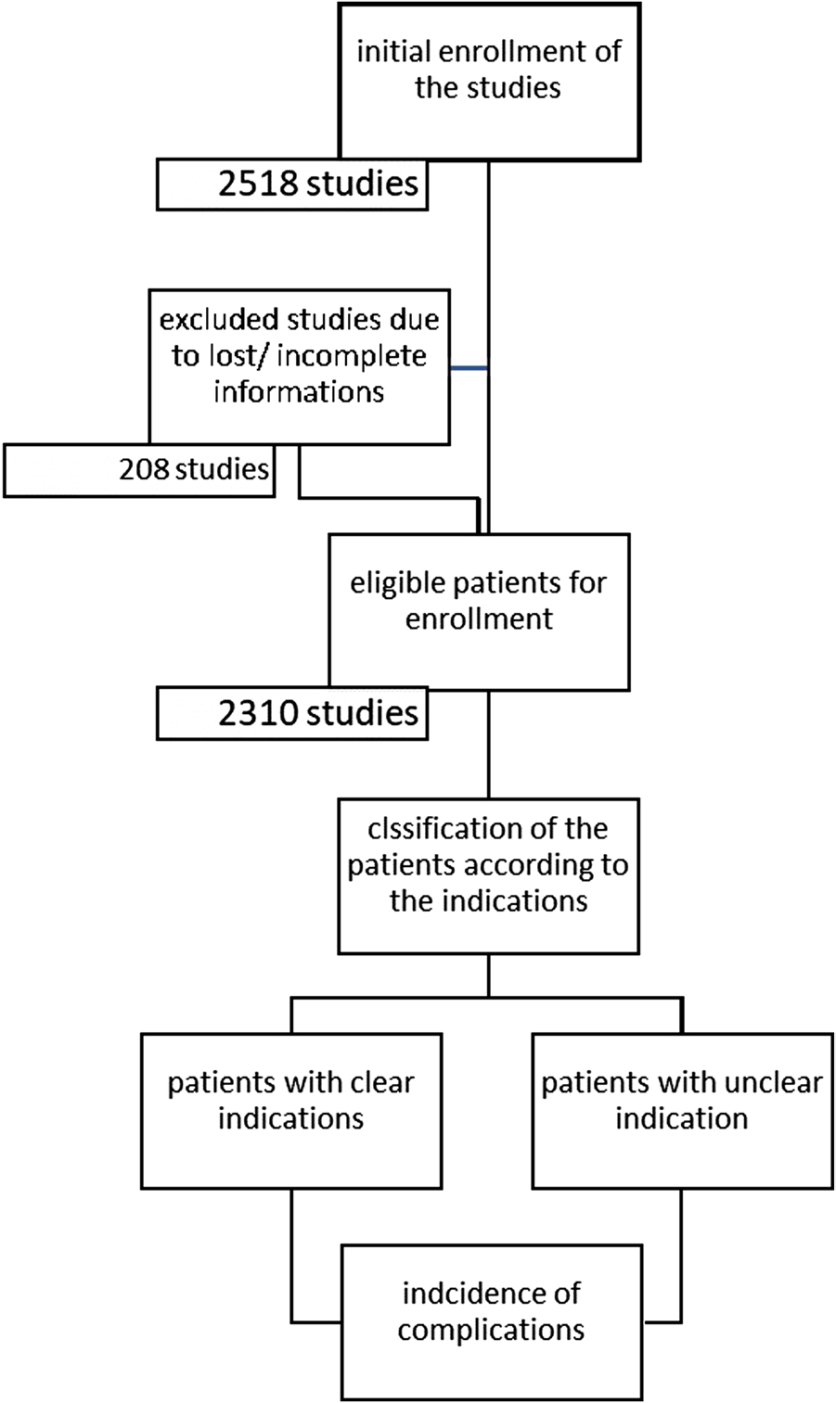
Patients enrolment in the study

This registry included a total 2518 patient who underwent MDCT in between 2016 and 2019. We excluded studies with incomplete, missed and lost data (Fig. 1).

The median age of the patients was 12 months (IQR 37 months), and the youngest patient was 3 days old. The eldest patient was 50 years old. 52.5% of our patients were males.

540 patients (23.37%) were in the neonatal period—first month of life. 1038 patients (44.9%) were in the infancy period (1 month–2 years).

Grown up patients with congenital heart disease (GUCH) more than 18 years old represented a minority of our patients’ population (3.6%).

1541 patients (66.7%) had congenital cyanotic heart disease. Almost half of these patients—772 patients (50%)—had a type of the tetralogy of Fallot disease spectrum either unoperated, post-shunt surgery or after total repair.

430 patients (18.6%) had previous surgical or transcatheter intervention (ex: arterial or venous shunt operation, total corrective surgery, transcatheter stent implantation, etc.) before the MDCT study.

### Analysis of the study population according to the target of the MDCT study showed that

#### Patients with septal defects for assessment of pulmonary veins

One hundred and ninety patients (8.9%) of the study population were sent to perform the MDCT study for the evaluation of pulmonary veins because of the suspicion of anomalous pulmonary venous drainage of cardiac, supra-cardiac, and infra-cardiac types (either partial anomalous pulmonary venous drainage, or total anomalous pulmonary venous drainage).

The most common referral diagnosis was patients with sinus venosus defects, followed by patients with ASD and suspected total anomalous pulmonary venous drainage of supra-cardiac type.

Anomalous pulmonary venous drainage was also found in association with other multiple complex congenital heart disease like left atrial and right atrial isomerism, and tetralogy of Fallot.

**Fig. 2:**
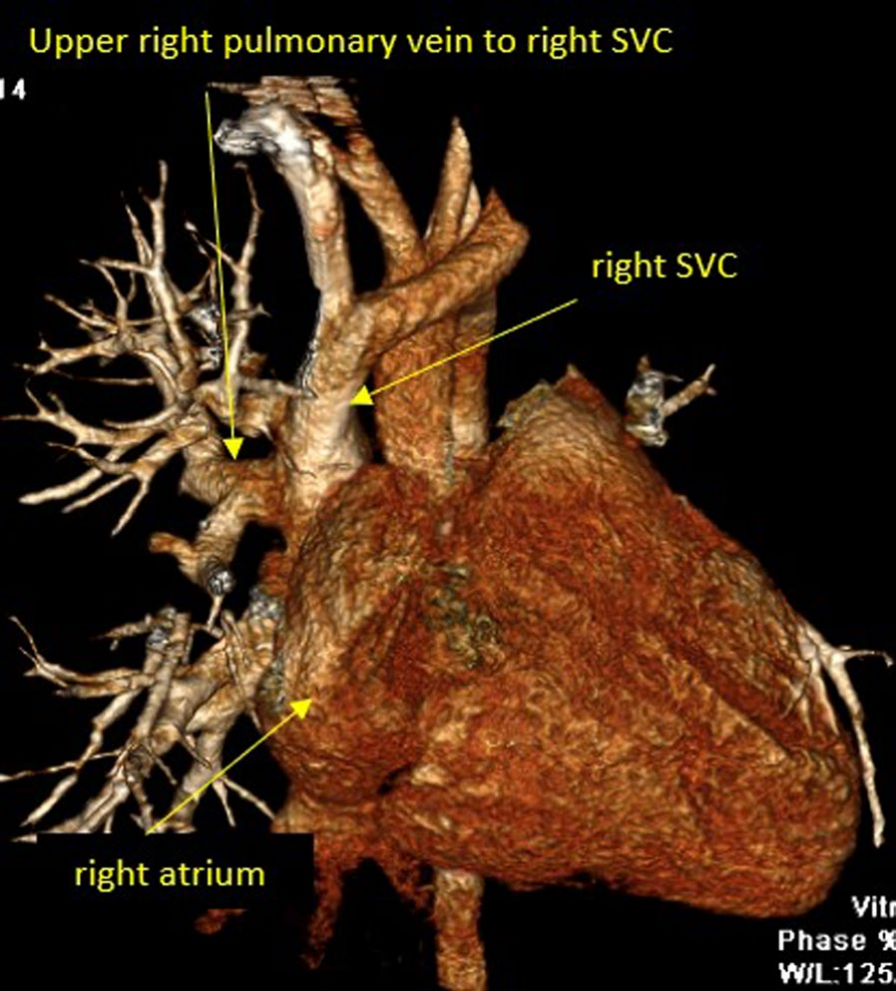
3D volume rendering image showing partial anomalous pulmonary venous drainage of the upper right pulmonary vein into the right superior vena cava

**Fig. 3:**
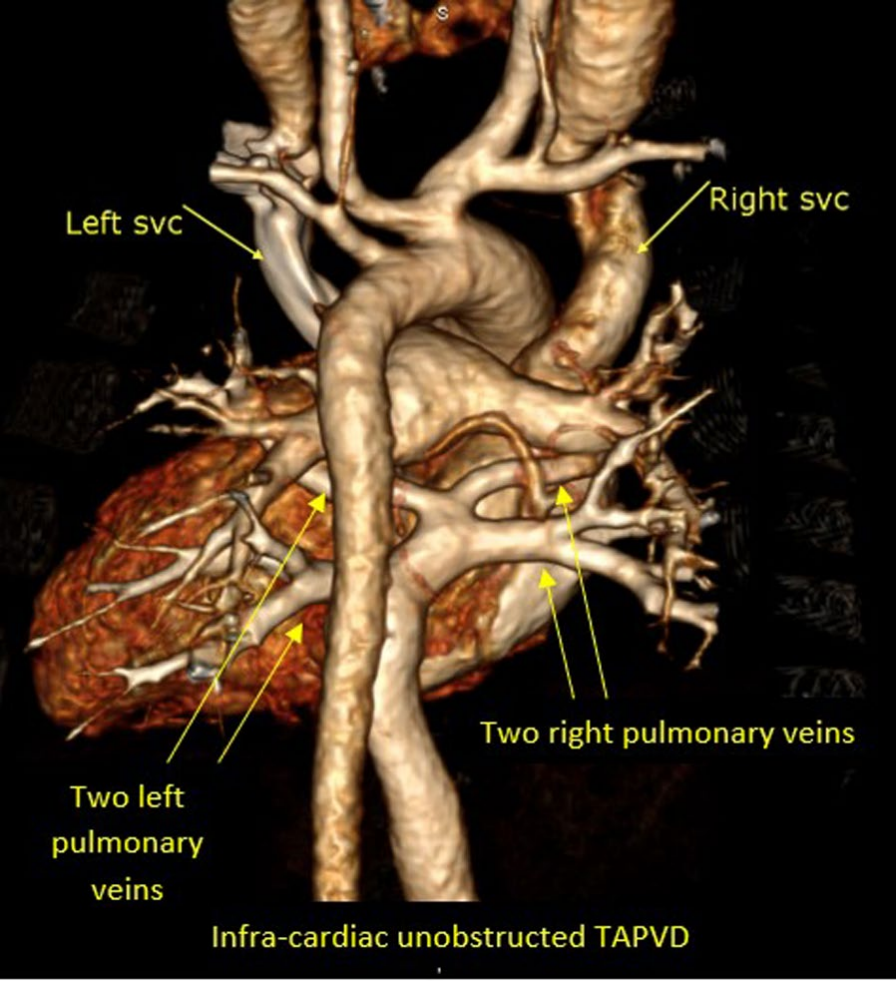
3D volume rendering image showing total anomalous pulmonary venous drainage unobstructed infra-cardiac type of the four pulmonary veins

**Fig. 4:**
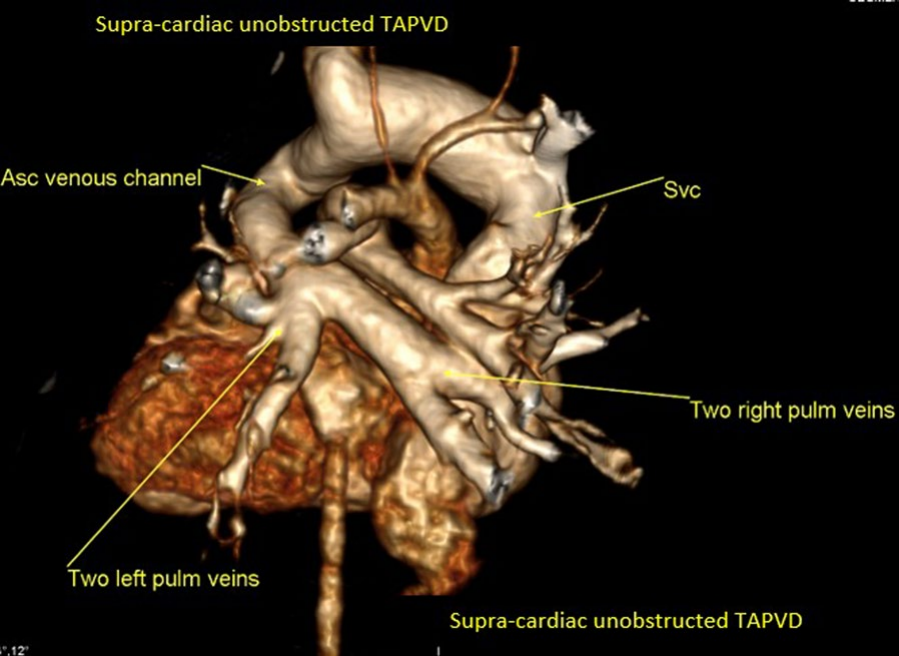
3D volume rendering image showing total anomalous pulmonary venous drainage unobstructed supra-cardiac type of the four pulmonary veins

Six patients in our registry (3.15%) had Scimitar syndrome. The MDCT ability to clearly visualize the course, site of drainage of the pulmonary veins and to identify any stenosis within their course was excellent, and the surgical details concurred with the MDCT descriptions in all of these patients (Figs. 2, 3, 4).

#### Patient’s with tetralogy of Fallot disease spectrum

Tetralogy of Fallot disease’s spectrum and variants was the most common congenital cyanotic heart disease in our registry. They represented almost half of the patients with congenital cyanotic heart disease 772 patients.

We included patients with common atrio-ventricular canal (CAVC) and Fallot’s tetralogy (canal Tet) in this group.

Eleven patients of the Fallot’s tetralogy disease group had partial anomalous pulmonary venous drainage of the left upper pulmonary vein into the left innominate vein representing 1.4% of theses patient.

**Fig. 5:**
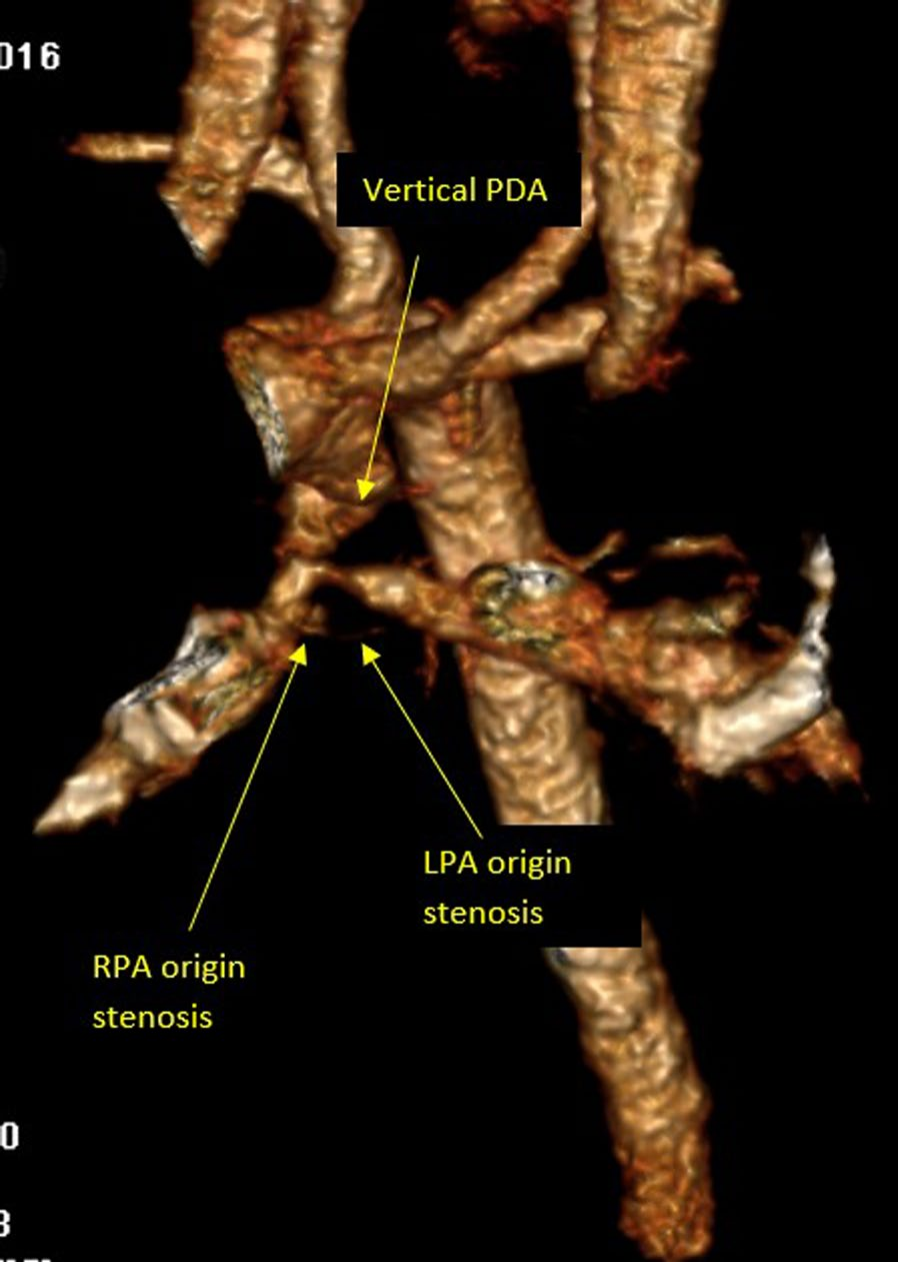
3D volume rendering image showing vertical PDA supplying hypoplastic pulmonary tree with bilateral pulmonary artery branches origin stenosis

Assessment of the pulmonary tree morphology, bifurcation pattern, and size, assessment of the major aorto-pulmonary collaterals (MAPCs), the morphology of the ductus arteriosus in cases of pulmonary atresia and coronary artery anatomy were the most common important targets in this group (Fig. 5).

#### Pulmonary atresia with intact interventricular septum

We had 15 patients with pulmonary atresia and intact interventricular septum, 5 of them were sent for MDCT after having their duct stented percutaneously. The incidence of right ventricular sinusoids dependent coronary circulation was 26.6% (4 patients).

#### Patent ductus arteriosus (PDA)

Assessment of the PDA as an isolated congenital heart disease was found in 33 patients (1.5% of the study cohort). This was done in young patients with PDA in associated with aortic coarctation, or adult patients with poor window and inability to assess the morphology, size, and the course of the duct.

#### Double outlet right ventricle (DORV)

Patients with double out right ventricle disease variables represented 155 patients (7.2%) of our registry, and the most common variant was the double outlet right ventricle, subaortic VSD, and severe pulmonary stenosis (65% of this group).

The main target of the study was the assessment of the pulmonary tree size and morphology, collaterals and coronary arteries. While the Taussig–Bing anomaly was present in only a minority of this group of patients and the target of the study was to exclude any associated aortic arch anomalies.

#### Congenitally corrected transposition of the great arteries

Patients with congenitally corrected transposition of the great vessels represented 20 patients (0.93%) in our cohort. Most of them had an associated ventricular septal defect and severe pulmonary stenosis.

We would like also to mention that 4 patients of them (20%) had an associated different degrees of congenital heart block.

#### Coronary artery anomalies

47 patients (2.2%) were referred to MDCT for pure evaluation of their coronary arteries.

**Fig. 6:**
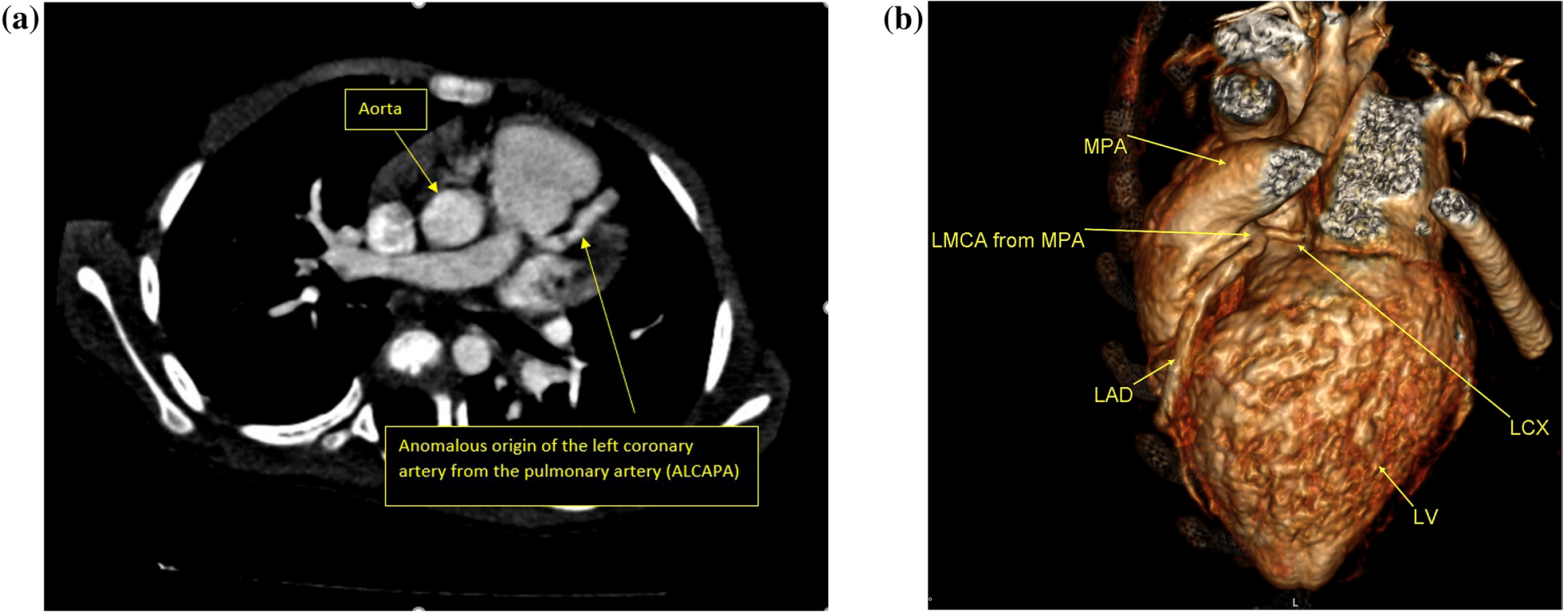
**a** Multiplanar axial image showing anomalous origin of the left pulmonary artery from the main pulmonary artery (ALCAPA syndrome), **b** 3D volume rendering image showing anomalous origin of the left pulmonary artery from the main pulmonary artery (ALCAPA syndrome)

The suspected referring diagnosis was congenital anomalies in the origin of the coronary arteries such as anomalous origin of the left coronary artery from the pulmonary artery (ALCAP syndrome (9 patients), coronary fistula (32 patients), and acquired disease as Kawasaki vasculitis (6 patients) (Fig. 6a, b).

#### Pulmonary artery aneurysm and peripheral pulmonary stenosis

Pulmonary artery aneurysm was the target of the study in 12 patients (0.56%). Peripheral pulmonary stenosis was the target of the study in 33 patients (1.5%); among these patients, the commonest syndrome was Alagille’s syndrome (6 patients). William’s Syndrome was the second common associated syndrome, but they are mentioned in the aortic group.

#### Assessment of surgical arterial shunts

Assessment of arterial shunt was performed in 150 patients.

This group included patients with Fallot’s tetralogy disease spectrum, or patients who has their shunt as the first stage of treatment of uni-ventricular repair, and other patients with two ventricles and associated severe pulmonary stenosis before their total corrective surgery.

**Fig. 7:**
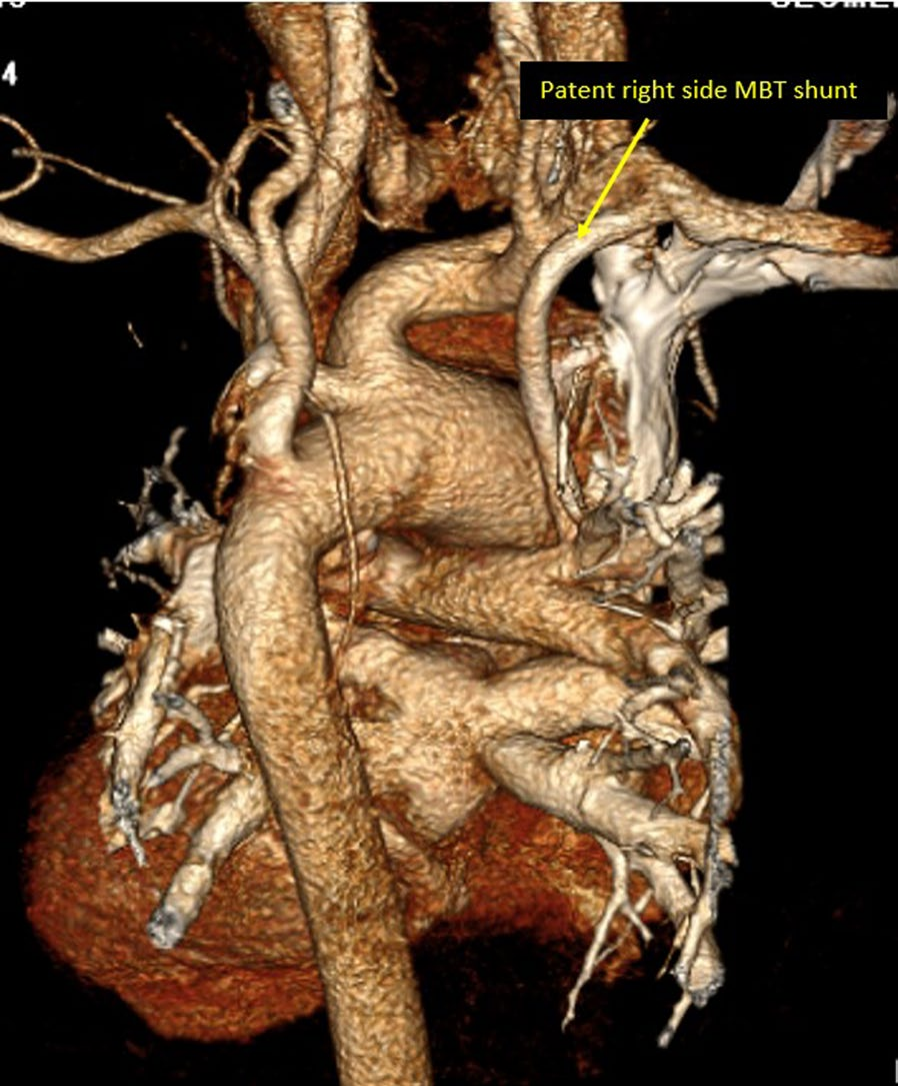
3D volume rendering image showing patent right MBT shunt from the right subclavian artery to the right pulmonary artery

Modified Blalock Taussig (MBT) shunt was the commonest arterial shunt among the studied cohort (145 patients), central shunt was found in minority of the patients (only 5 patients). It is worth mentioning that 43 patients had bilateral MBT shunts (Fig. 7).

#### Assessment of cavo-pulmonary shunts (Glenn’s and Fontan’s shunt)

Assessment of venous shunts (Glenn, Fonatain, and venous collaterals) was the target in 185 patients (8.6%); among these patients, 28 patients had bilateral bidirectional Glenn shunt.

#### Assessment of atrial baffles (in patients with atrial switch operations)

Transposition complexes with Atrial switch (Mustard or Senning Procedure) represent only 5 patients in our study, and assessment of the baffle was the main target of the study. We had no patients in our study post-arterial switch.

#### Assessment of right ventricle to pulmonary artery conduit (RV-PA conduit)

**Fig. 8:**
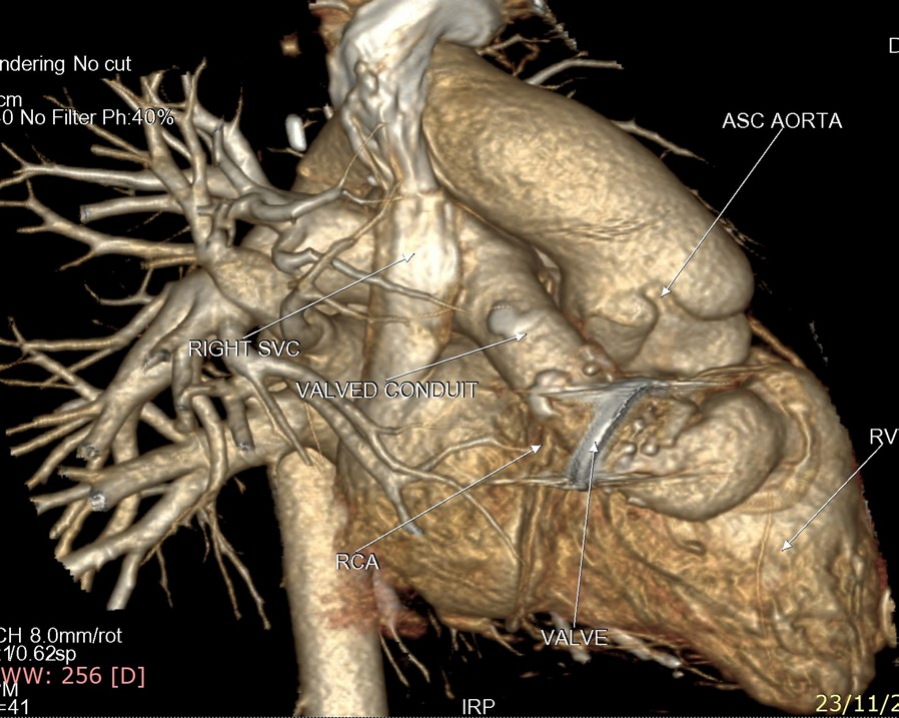
3D volume rendering image showing valved conduit from the right ventricle to the main pulmonary artery with mild calcification

Patients with RV-PA conduit represented 33 patients in our registry. Rastelli’s operation was the most common operation among those patients (Fig. 8).

#### Patients with single ventricle

Single ventricle heart disease patients were seen in our cohort very early either before or after stage 1 surgery (systemic to pulmonary arterial shunt), After stage 2 surgery (Glenn or Hemi-Fontan procedure), only one patient in our cohort was seen after stage 3 surgery (Fontan’s shunt).

#### Aortic disease, truncus arteriosus, aorto-pulmonary window, rupture sinus of Valsalva, collagen, and connective tissue syndromes and diseases

**Fig. 9:**
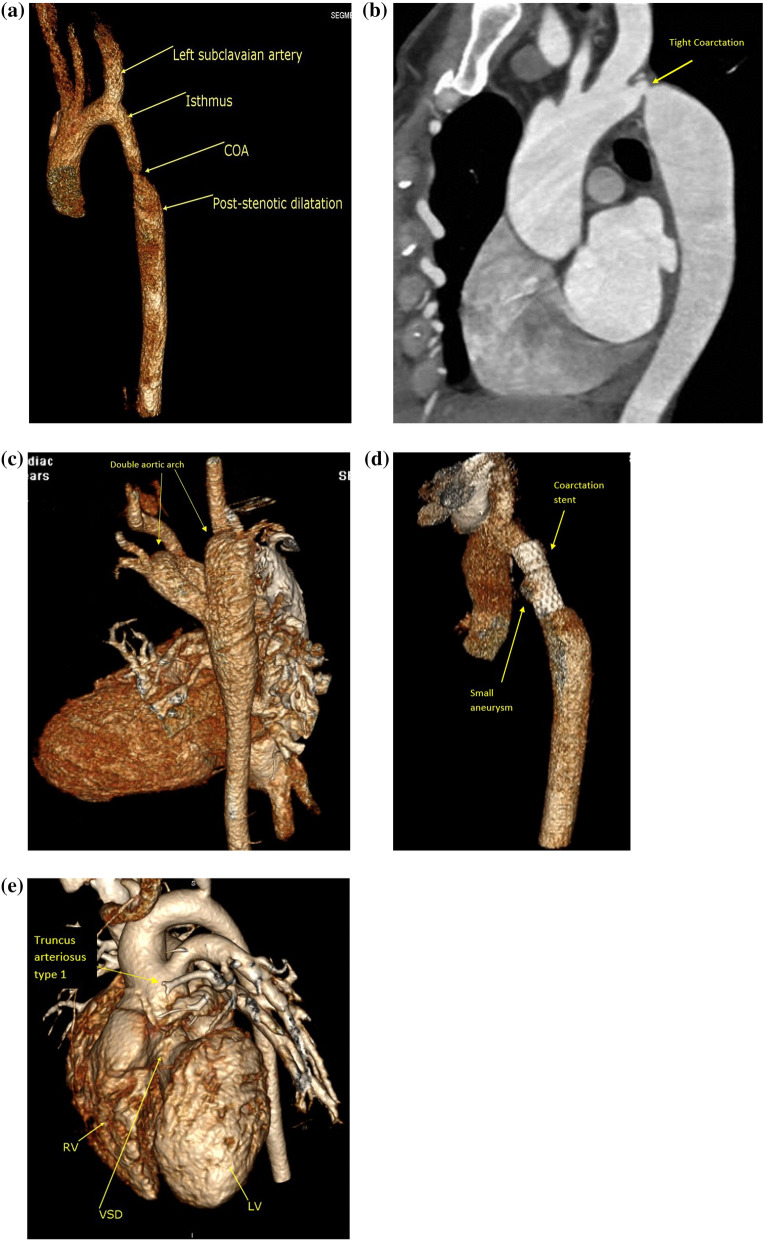
**a** 3D volume rendering image showing tight focal aortic coarctation. **b** multiplanar sagittal image showing tight focal coarctation immediately distal to the left subclavian artery. **c** 3D volume rendering image showing double aortic arches (complete vascular ring). **d** 3D volume rendering image showing well deployed expanded stent with starting aneurysm within its mid segment. **e** 3D volume rendering image showing truncus arteriosus type 1

Assessment of aortic disease like bicuspid aortic valve associated aortopathy (60 patients), native coarctation (280 patients), post-coarctation surgical repair (23 patients), post-coarctation stenting (19 patients), aortic interruption (16 patients), complete and incomplete vascular rings (40 patients), truncus arteriosus (28 patients), aortopulmonary window (20 patients), supravalvular aortic stenosis (21 patients), arterial tortuosity syndrome (2 patients), Loeys–Dietz syndrome (1 patient), Ehlers–Danlos syndromes (1 patient), Marfan syndrome (4 patients), 3 patients with rupture sinus of Valsalva (Fig. 9a–e).

#### Surgical re-entry high risk patients

Sternal re-entry in high-risk patients was the indication in 22 patients. All had previous surgical palliative or total repair procedures. Fallot’s tetralogy total repair, severe pulmonary regurgitation, and dilated right ventricular outflow tract that was adherent to the sternal surface were the most common indication. Supravalvular aortic stenosis with pseudoaneurysm, aneurysm or recurrence was the second most common indication.

The study was done to plan for surgical re-entry before reconstruction of the right ventricular outflow tract, pulmonary valve implantation, or aortic repair.

#### Topsy Turvy syndrome

**Fig. 10:**
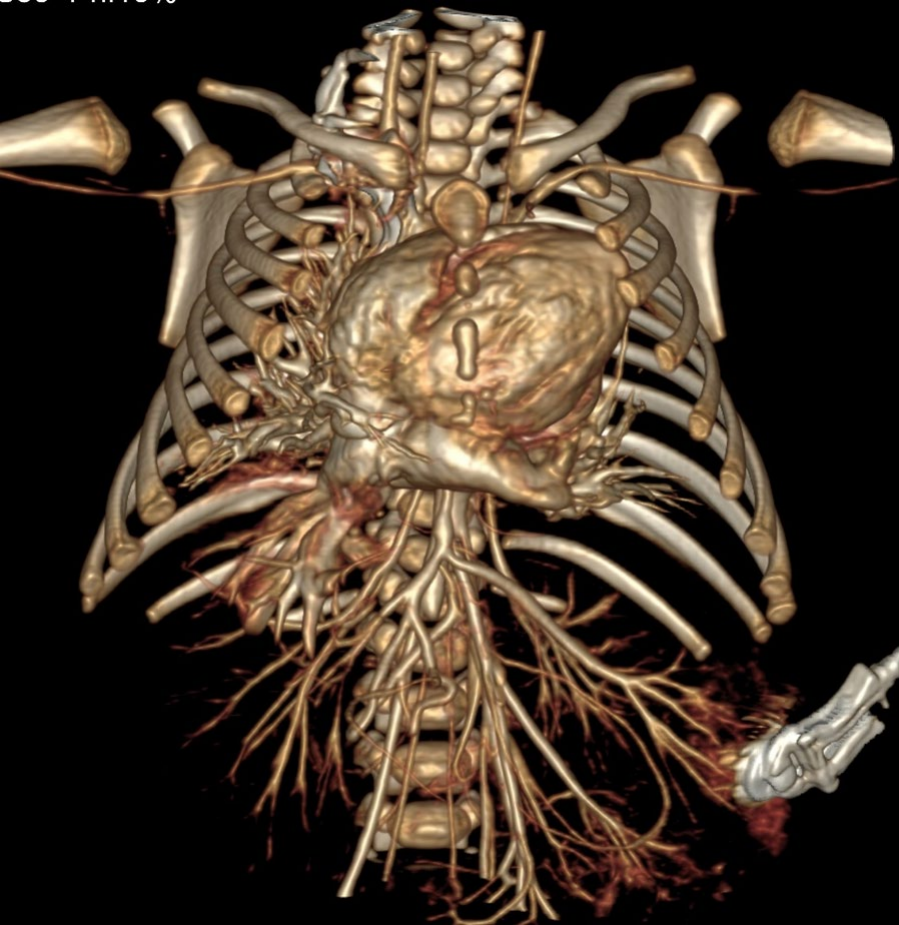
3D volume rendering image showing Topsy Turvy heart with the cardiac apex in the left infraclavicular are

We had one case with Topsy Turvy heart syndrome (Fig. 10).

#### Overlap between groups

Patients with right and left atrium isomerism were present in all the mentioned groups, according to the predominant target of the study and associated intracardiac lesions.

Patients with functionally single ventricle and univentricular repair were mentioned in details in the sections of arterial, and venous shunts.

#### Controversial and/or inappropriate indications

Controversial and/or inappropriate indications for MDCT were found in 30 patients (1.4%). In this group, cardiac catheterization, direct surgical intervention, other investigation like echocardiography with bubble study were deemed by us to be the next step or next investigation of choice and MDCT results did not add significant value for the evaluation of the patients, deeming the selection of the MDCT study to be rather inappropriate. 10 patients had mild pulmonary stenosis with associated subaortic membrane, 5 patients had ventricular septal defect and PDA, 8 patients had dilated coronary sinus with suspected persistent left SVC, 4 patients had DORV, VSD, unprotected pulmonary circulation, and 3 patients had suspected VSD.

#### Declining the study

We have declined doing the MDCT for three patients in this registry.

All had an associated congenital pelvi-uretric junction obstruction, and/or posterior ureteral valve with recurrent urinary tract infections, hydroureter, hydronephrosis, and elevated kidney function test with reduced glomerular filtration rate. After consulting with their referral physicians, they were referred to do other investigation such as cardiac MRI, or they were referred directly to surgery.

#### Diagnostic accuracy of the study

The ability of the MDCT study to accurate diagnose or exclude the patient’s condition was assessed by feedback and communication with the referral center, surgeon, and cardiologist.

The diagnostic yield excellent based on either intraoperative finding and data of the patients who underwent total and palliative procedures, or patient who underwent invasive cardiac catheterization.

Missing of the diagnosis and or missing data was encountered in two patients (less than 1 %) in this registry, and two of them had anomalies of the coronary artery origin with contradicting clinical and MDCT report data, where MDCT missed the diagnosis of anomalies origin of the left main coronary artery from the pulmonary artery (ALCPA) syndrome due to poor image quality of the study, rapid heart rate of the patient combined with motion and breathing artifacts. The diagnosis was confirmed by invasive catheterization in this patient.

The second patient had incomplete assessment of the course and termination of coronary artery fistula from the left circumflex artery to the right ventricle basal inferior wall near the coronary sinus entry, and it was also delineated by invasive catheterization.

### Analysis of the incidence of the procedure-related complication

#### Venous access data and access-related complications

Intravenous access was obtained in the upper limb in 56.7% of the patients, in the lower limb in 13.5% of the patients, in the neck veins in 23.5% of the patients, in the scalp veins in 4.6% of the patients, anterior abdominal wall in 1.4% of the patients. Simultaneous accesses in the upper and lower halves of the body were obtained in less than 1% of the patients.

Difficulty in obtaining the venous access with more than three trials to insert the venous cannula was encountered in 3% of the patients. Postponing the study due to failure to obtain the venous access was encountered very rarely in less than 0.5% of the patients.

Twelve patients—0.51% of the patients—had extravasation of the contrast material in the subcutaneous tissue due to rupture of the veins, with mild erythema and subcutaneous edema. Immediate interruption of contrast material and/or saline injection was done as the pressure tracing showed abrupt rise or the patient reported pain during injection.

The volume of the contrast material delivered to the circulation and motion artifacts secondary to pain from the extravasated contrast rendered the study uninterpretable with very poor images in only 5 patients of this group, and the study was repeated after securing another venous access.

The image quality was satisfactory in the other 7 patients with no need to repeat the study.

Extravasation of the contrast material and vein rupture was managed conservatively in all of the patients with warm fomentation, skin care, and local application of anti-inflammatory, antiedema creams. None of these patients developed delayed complication like skin ulceration or required any further management.

#### Allergic reactions

Twenty-four patients—1.03% of the study population—had allergic reactions from contrast material injection. All reactions were minor (skin erythema, wheals, and itching); they were managed by antihistaminic. We did not encounter any severe forms of allergic reactions among our study population ex: stridor with bronchospasm and wheezes or anaphylaxis, etc.

#### Side effects of anesthesia

Transient apnoea and bradycardia were the most common encountered side effects in 3.1% and 2.4% of the patients, respectively, with uneventful continuation of the study.

Hiccough and vomiting were less frequent. Hiccough lead to some difficulty in interpretation of the study images due to the sudden high amplitude motion artifacts. We used the ECG gating to perform multiple reconstructions of the images, and none of these patients needed to repeat the study.

Agitation and delayed recovery from anesthesia were only rarely encountered.

#### Cyanotic spells

Cyanotic spell occurred in seven patients just after administration of anesthesia or immediately after the end of the study. All of the patients were successfully managed according to the standardized protocols without any cardiac or neurological complications. And the study was completed successfully.

#### Mortality

Unfortunately, we have encountered one case of mortality 0.04% that occurred 3 days after the study. This patient had multisystem congenital anomalies, dysmorphic features with neurological malformation. He had apnoea, bradycardia just after the study. He was successfully resuscitated, intubated, and transferred to the NICU; however, his condition deteriorated furtherly.

## Discussion

Our study represents the first documented MDCT registry for patients with congenital heart disease in our country.

The purpose of any MDCT study in patients with congenital heart disease is addressing a certain unconfirmed question related to mainly extracardiac and occasionally intracardiac structures.

The majority of our registry’s population were neonates and young children less than 2 years of age, and complex congenital heart diseases were the commonest diagnosis in them.

The majority of patients had cyanotic heart disease with different variants of Fallot’s tetralogy being the commonest cyanotic heart disease to be a cause for MDCT referral.

Our registry population were referred to do MDCT examination within their various disease’s stages, either initially for diagnosis and delineation of anatomy, or after staged procedure, or after their final corrective surgery.

MDCT played an important role in surgical and transcatheter intervention planning, and it was also used in evaluation and follow-up after different surgical and transcatheter interventions.

MDCT study proved to be a fast, convenient, safe, and cost-effective tool. It has high spatial and temporal resolution and the advantage of 3D images reconstruction.

The use of recent protocols allowed us to perform the study with good images quality and minimizing the dose of radiation.

The incidence of procedural related complications was very low, and most of these complications were minor, and non-life threatening.

## Conclusions

We concluded that the majority of patients with congenital heart disease who were referred for multidetector computed tomography study in our country between 2016 and 2019 were infants and young children with complex cyanotic heart disease. The study was performed with high diagnostic accuracy using the body weight-adjusted contrast volume, high safety, and low incidence of complications. Vascular Access complications were the most common encountered problem. MDCT is a fast safe and reliable imaging modality in diagnosis of complex congenital heart disease and extra cardiac anomalies.

## Data Availability

The datasets used and/or analyzed during the current study are available from the corresponding author on reasonable request.

## Abbreviations

ASD: Atrial septal defect
VSD: Ventricular septal defect
PDA: Patent ductus arteriosus
CAVC: Common atrioventricular canal
PAPVD: Partial anomalous pulmonary venous drainage
MDCT: Multidetector computed tomography
ECG: Electrocardiogram
DORV: Double outlet right ventricle
MBT: Modified Blalock–Taussig shunt
NICU: Neonatal intensive care unit
ALCAP: Anomalous origin of the left coronary artery from the pulmonary artery
RV: Right ventricle
PA: Pulmonary artery
MAPCs: Major aorto-pulmonary collaterals
GUCH: Grown up congenital heart disease
